# A world view: Considerations of sociocultural diversity in the study of subjective cognitive decline in Alzheimer's disease and related dementias

**DOI:** 10.1002/alz.71779

**Published:** 2026-09-02

**Authors:** Mark A. Dubbelman, Tanisha G. Hill‐Jarrett, Silvia Chapman, Gustavo Alves Andrade dos Santos, Martina Azar, Ganesh M. Babulal, Tyler R. Bell, Mark A. Bernard, Carolina Boza‐Calvo, Anthony Q. Briggs, Omonigho Michael Bubu, Heather E. Cuevas, N. Maritza Dowling, Darlingtina K. Esiaka, Bernadette A. Fausto, Fabricio Ferreira de Oliveira, Carol E. Franz, Joyla A. Furlano, Christopher Gonzalez‐Corona, M. Aaron Guest, Veer Bala Gupta, Vivek K. Gupta, Daija Jackson, Jillian L. Joyce, Michael R. Kann, William S. Kremen, Athene Lee, Jairo E. Martinez, Arjun V. Masurkar, Maia A. McLin, Elisa de Paula França Resende, Zahra Rahemi, Brianca C. Renfro, Márlon Juliano Romero Aliberti, Shaina Shagalow, Andrea Slachevsky, Juliana N. De Souza Talarico, Jean François Trani, Carol Van Hulle, Leah Waltrip, Stephen Ojiambo Wandera, Charles C. Windon, Mônica Sanches Yassuda, Davide V. Moretti, Elizabeth Kuhn, Elke Butterbrod, Katherine A. Gifford, Sietske A. M. Sikkes, Raphael M. Castilhos, Rachel Nosheny, Shana D. Stites, a collaboration of the Alzheimer's Association International Society to Advance Alzheimer's Research and Therapy Subjective Cognitive Decline and Diversity and Disparities Professional Interest Areas

**Affiliations:** ^1^ Center for Alzheimer Research and Treatment Department of Neurology Harvard Medical School Brigham and Women's Hospital Boston Massachusetts USA; ^2^ Department of Neurology Harvard Medical School Massachusetts General Hospital Boston Massachusetts USA; ^3^ Department of Neurology, Memory and Aging Center University of California San Francisco San Francisco California USA; ^4^ Global Brain Health Institute University of California San Francisco San Francisco California USA; ^5^ Cognitive Neuroscience Division, Taub Institute for Research on Alzheimer's Disease and the Aging Brain Columbia University Irving Medical Center New York New York USA; ^6^ Department of Anatomy and Surgery Ribeirão Preto Medical School University of São Paulo Ribeirão Preto Brazil; ^7^ Department of Food Science and Nutrition School of Food Engineering University of Campinas Campinas Brazil; ^8^ São Leopoldo Mandic School of Medicine São Paulo Brazil; ^9^ Brown University Health Providence Rhode Island USA; ^10^ Brown University Warren Alpert Medical School Providence Rhode Island USA; ^11^ Department of Neurology Washington University School of Medicine St. Louis Missouri USA; ^12^ Institute of Public Health Washington University in St. Louis St. Louis Missouri USA; ^13^ Centre for Social Development in Africa Faculty of Humanities University of Johannesburg Johannesburg South Africa; ^14^ Department of Psychiatry University of California San Diego San Diego California USA; ^15^ Center for Behavioral Genetics of Aging University of California San Diego San Diego California USA; ^16^ Department of Neurology, New York University Grossman School of Medicine New York New York USA; ^17^ Department of Population Health New York University Grossman School of Medicine New York New York USA; ^18^ School of Nursing, The University of Texas at Austin Austin Texas USA; ^19^ School of Nursing George Washington University Washington District of Columbia USA; ^20^ Milken Institute School of Public Health Department of Biostatistics & Bioinformatics George Washington University Washington District of Columbia USA; ^21^ Center for Health Engagement and Transformation. University of Kentucky Lexington Kentucky USA; ^22^ Department of Behavioral Science University of Kentucky College of Medicine Lexington Kentucky USA; ^23^ Sanders Brown Center on Aging University of Kentucky College of Medicine Lexington Kentucky USA; ^24^ Center for Molecular & Behavioral Neuroscience Rutgers University–Newark Newark New Jersey USA; ^25^ Department of Health Science & Clinical Practice Thomas Jefferson University Philadelphia Pennsylvania USA; ^26^ Escola Paulista de Medicina, Federal University of São Paulo São Paulo Brazil; ^27^ Department of Family Medicine, Queen's University Kingston Ontario Canada; ^28^ Rosalind Franklin University of Medicine and Science Chicago Illinois USA; ^29^ Center for Innovation in Healthy and Resilient Aging, Edson College of Nursing and Health Innovation, Arizona State University Phoenix Arizona USA; ^30^ School of Medicine, Deakin University Geelong Victoria Australia; ^31^ Faculty of Medicine and Health Sciences, Macquarie University Sydney New South Wales Australia; ^32^ College of Graduate and Professional Studies, The Chicago School Chicago Illinois USA; ^33^ Leonard Davis School of Gerontology, University of Southern California Los Angeles California USA; ^34^ University of Pittsburgh School of Medicine, University of Pittsburgh Pittsburgh Pennsylvania USA; ^35^ Department of Psychiatry and Human Behavior Brown University Warren Alpert Medical School Providence Rhode Island USA; ^36^ Butler Hospital Providence Rhode Island USA; ^37^ Department of Psychological and Brain Sciences Boston University Boston Massachusetts USA; ^38^ Department of Psychiatry Massachusetts General Hospital Harvard Medical School Boston Massachusetts USA; ^39^ Department of Neuroscience New York University Grossman School of Medicine New York New York USA; ^40^ Department of Psychology Mississippi State University Mississippi State Mississippi USA; ^41^ Faculdade de Medicina Universidade Federal de Minas Gerais Belo Horizonte Brazil; ^42^ Faculdade de Medicina Faculdade de Ciências Médicas de Minas Gerais Belo Horizonte Brazil; ^43^ School of Nursing, Clemson University Greenville South Carolina USA; ^44^ Department of Psychiatry, Geriatric Center for Cognitive Medicine, Vanderbilt University Medical Center, Vanderbilt University Nashville Tennessee USA; ^45^ Division of Geriatrics University of São Paulo Medical School São Paulo Brazil; ^46^ Sirio‐Libanes Research Institute São Paulo Brazil; ^47^ Department of Neurology, Division of Cognitive Neuroscience Columbia University Medical Center New York New York USA; ^48^ Gerosciences Center for Brain Health and Metabolism Santiago Chile; ^49^ Memory and Neuropsychiatric Center Neurology Department, Hospital del Salvador, Faculty of Medicine University of Chile Santiago Chile; ^50^ Neuropsychology and Clinical Neuroscience Laboratory (LANNEC) Interdisciplinary Nucleus for Physiology, Biophysics and Pathophysiology Institute of Biomedical Science Neuroscience and East Neuroscience Departments Faculty of Medicine University of Chile Santiago Chile; ^51^ Neurology and Psychiatry Department Clínica Alemana University Desarrollo Santiago Chile; ^52^ College of Nursing University of Iowa Iowa City Iowa USA; ^53^ Conservatory of Arts and Crafts Paris France; ^54^ Department of Psychology University of Johannesburg Johannesburg South Africa; ^55^ Department of Medicine School of Medicine and Public Health University of Wisconsin–Madison Madison Wisconsin USA; ^56^ Alzheimer's Disease Research Center School of Medicine and Public Health University of Wisconsin–Madison Madison Wisconsin USA; ^57^ Department of Psychology, University of Denver Denver Colorado USA; ^58^ Department of Population Studies, School of Statistics and Planning, College of Business and Management Sciences, Makerere University Kampala Uganda; ^59^ Gerontology School of Arts Sciences and Humanities University of São Paulo São Paulo Brazil; ^60^ Department of Neurology School of Medicine University of São Paulo São Paulo Brazil; ^61^ IRCSS Istituto Centro San Giovanni di Dio Fatebenefratelli Alzheimer Rehabilitation Operative Unit Brescia Italy; ^62^ Normandie University, UNICAEN, INSERM Caen France; ^63^ German Center for Neurodegenerative Diseases (DZNE) Bonn Bonn Germany; ^64^ Department of Old Age Psychiatry and Cognitive Disorders University Hospital Bonn Bonn Germany; ^65^ Department of Clinical Neuro‐, and Developmental Psychology, Faculty of Movement and Behavioral Sciences Vrije Universiteit Amsterdam Amsterdam the Netherlands; ^66^ Alzheimer Center Amsterdam Department of Neurology Amsterdam University Medical Center Amsterdam the Netherlands; ^67^ Department of Anatomy & Neurobiology, Boston University Chobanian & Avedisian School of Medicine Boston Massachusetts USA; ^68^ Cognitive and Behavioral Neurology Center Neurology Service Hospital de Clínicas de Porto Alegre Porto Alegre Brazil; ^69^ Departamento de Medicina Interna Universidade Federal do Rio Grande do Sul Porto Alegre Brazil; ^70^ Department of Psychiatry and Behavioral Sciences University of California San Francisco San Francisco California USA; ^71^ Department of Radiology and Biomedical Imaging University of California San Francisco San Francisco California USA; ^72^ Northern California Institute for Research and Education San Francisco California USA; ^73^ Department of Psychiatry and Division of Geriatric Medicine in the Department of Medicine Perelman School of Medicine University of Pennsylvania Philadelphia Pennsylvania USA

**Keywords:** Alzheimer's disease, Alzheimer's disease and related dementias, cognitive concerns, diversity, sample representation, subjective cognitive decline

## Abstract

Subjective cognitive decline (SCD) refers to cognitive concerns that may occur with or without objective impairment on standardized testing. Studies suggest that SCD may be an early clinical marker of Alzheimer's disease and related dementias, and that it can predict future objective cognitive decline and progression to dementia. However, the research that supports these conclusions is primarily based on samples with homogeneous socioeconomic and cultural backgrounds. The limited studies in samples with more diverse representations of racial, ethnic, gender, and sexual orientation characteristics show varying SCD prevalence, correlates, and outcomes. Here, we review this literature, focusing on how to apply the findings to advance our understanding of SCD. We further evaluate how our findings inform a roadmap for future research. Overall, we conclude that broader investigations across more diverse populations enhance our understanding of the factors that may differentially contribute to SCD and shape its utility as a clinical metric.

## Highlights

- Subjective cognitive decline (SCD) may be an early sign of Alzheimer's disease.
- SCD research is largely based on homogeneous samples with limited representation.
- This review describes SCD research from samples with diverse representations.
- SCD correlates, outcomes, and prevalence vary with aspects of diversity.
- Diverse studies enhance our understanding of SCD's utility as a clinical metric.

## 1 BACKGROUND

Subjective cognitive decline (SCD) typically refers to a self‐perceived deterioration in cognition from a previous level, often in the absence of cognitive impairment on objective neuropsychological testing.^1^ More broadly, SCD can also refer to cognitive concerns in general. In some situations, SCD can be one of the earliest clinical manifestations of Alzheimer's disease (AD) and related dementias (ADRD).^1^, ^2^ As such, SCD has been conceptualized as an early clinical stage of ADRD. The recently updated clinical staging criteria for AD from the US National Institute on Aging and Alzheimer's Association (NIA‐AA) have included SCD among amyloid‐positive individuals as a possible first clinical feature of ADRD (“stage 2” in amyloid‐positive individuals), thereby cementing the consensus regarding its utility in determining the future risk of dementia.^3^ At the same time, SCD has historically been attributed to psychiatric symptoms, which arise from functional disturbances in mood, behavior, and thought, rather than to neurodegenerative disease.^4^, ^5^, ^6^, ^7^ More recently, emerging data are raising the possibility that SCD, like agitation, anxiety, and depression, may in fact reflect neuropsychiatric symptoms. Thus, SCD could reflect biological changes associated with AD,^8^, ^9^, ^10^, ^11^, ^12^ but it can also emerge in other neurodegenerative or healthy aging‐related processes. Regardless of the role SCD might play in neurodegenerative disease, SCD has been linked to diminished well‐being and quality of life in older adults, warranting its examination and addressing as a pressing clinical issue.^13^, ^14^, ^15^

It is well known that sociodemographic characteristics like race, ethnicity, sex, gender identity, sexual orientation, education, socioeconomic status, and cultural background influence clinical outcomes, including SCD.^16^, ^17^, ^18^ However, as is the case in much of the biomedical research at large, most work published on SCD relies primarily on data from Western, highly educated, industrialized, rich, and democratic samples. To accurately characterize SCD, understand its mechanisms, and address it in ways that improve patient well‐being, we need a clearer understanding of how social, cultural, psychological, and identity‐related factors affect SCD. Investigating SCD across diverse populations will help clarify how these factors shape the subjective experiences of SCD, inform how it may manifest in different contexts, and help identify factors that may signal increased vulnerability to ADRD. Without adequately considering social, cultural, psychological, and identity‐related factors, current models risk relying on interpretative frameworks derived from limited populations, potentially overlooking important variability in how early cognitive changes are experienced and reported across the world.

Studying heterogeneous populations can help limit the effects of sample biases and improve rigor in the assessment of the SCD construct by showing the construct's invariance, or lack thereof, across diverse sociocultural groups. Considering sociocultural context further facilitates the integration of structural and social determinants of health, which are known to strongly influence health outcomes and health‐care access.^19^ This perspective is particularly relevant for SCD, as these determinants may shape both the reporting of cognitive concerns and their associations with future cognitive decline and health‐care use.

One factor that complicates the study of SCD across diverse populations is the inconsistent terminology used in the literature. Consequently, it is imperative to define both “SCD” and “diverse populations” for the purposes of this review. In the literature we cite, SCD is sometimes used restrictively to refer to the phenomenon of having cognitive concerns without objective impairment. Here, however, we use the term SCD to refer broadly to cognitive concerns perceived by individuals and/or their proxies (e.g., spouses, relatives, friends) that may occur in the absence or presence of (subtle) objective impairment on standardized cognitive testing. This deliberate choice was made to avoid excluding research from contexts in which cognitive testing is not readily available and to acknowledge that, for individuals experiencing SCD, the clinical determination of cognitive impairment is inconsequential to their subjective experience of a serious, life‐impacting problem. As for “diversity,” we use this term in its broadest sense, encompassing many aspects in which groups of people can differ from one another, including, but not restricted to, race, ethnicity, ancestry, sex, cultural background, sexual orientation, gender identity, geography, linguistic background, and sociopsychological and socioeconomic status. When referring to the totality of these factors, we will use the term “aspects of diversity,” and otherwise, we will refer to the specific aspect under discussion.

In this narrative review, we offer a synthesis of existing studies to identify insights, gaps, and priorities for future studies. First, we discuss how diversity in SCD measurement may influence conclusions about its clinical utility across populations. Second, we delve into research on aspects of diversity and their influences on the relationship between SCD and ADRD. We discuss the implications of the ADRD field's shift in focus toward using SCD as an early clinical marker of ADRD and evaluate how past findings inform a roadmap for future research. Next, we address the influence of social, structural, psychological, and cultural factors on SCD. Finally, we cover lessons learned, future directions, and recommendations to make the study of SCD more inclusive and ultimately, less biased by selective representation.

## 2 DIVERSITY IN THE MEASUREMENT OF SCD

### 2.1 Measurement properties might amplify bias

Researchers and clinicians have myriad ways to assess the presence and severity of SCD, ranging from elaborate, well‐validated questionnaires to ad hoc single questions. Although there have been efforts to harmonize SCD questionnaires to establish the measurement of an underlying construct representing SCD,^20^ in practice, there are several sources of inconsistency in SCD measurement, three of which we will discuss below.

First, there is structural heterogeneity in questions assessing SCD. Many researchers and clinicians use a simple yes/no question to determine whether individuals meet a criterion for a self‐reported experience that is consistent with SCD (e.g., asking participants simply whether they have any concerns about their memory). In studies using single‐item SCD measures, the sensitivity for predicting conversion to mild cognitive impairment (MCI) and dementia (i.e., correctly identifying individuals who will progress to MCI or dementia) ranged from 26% to 96%.^21^ The specificity of single‐item SCD measures (i.e., the ability to correctly identify individuals who will not progress) ranged between 45% and 100%.^21^ This wide range of accuracy is likely due to significant variability in the structure, administration, and error rates of single‐item measures (e.g., sample selection).^21^ Single‐item measures generally do not assess the severity or domain specificity of a criterion for self‐reported SCD.^22^ By relying on a single item, measures may lead to more pronounced biases across factors such as race, ethnicity, and sex or gender identity, particularly when respondents have a wider range of interpretations. Studies have also shown that single‐item measures are highly confounded by a heritable trait‐like factor that reflects long‐standing differences in mood and personality rather than AD risk.^23^ In fact, psychometric standards recommend that no fewer than three items comprise a facet of evaluation.^24^ Cross‐cultural studies generally have shown that single‐item measures offer tremendous efficiency in large‐scale studies (e.g., World Values Survey) and offer decent validity for simple constructs like life satisfaction. Yet, for nuanced constructs like SCD, they risk lower reliability and greater measurement error than multi‐item scales. They are also prone to cultural differences in language interpretation and response biases, such as extreme response styles or modesty biases.

Content heterogeneity also affects measurement. A review of 19 studies using SCD measures revealed that most scales assessed a single cognitive domain (most commonly memory), while some assessed multiple domains of cognitive function.^22^ SCD is not purely a memory‐related concern and may not show a one‐to‐one correspondence with well‐recognized neuropsychological or cognitive domains.^25^ When someone is probed in too restrictive a manner, they might not report other concerns that are not explicitly asked about. It is conceivable that aspects of diversity that affect a person's lived experiences can condition what a person reports as a correlate of membership in a social group. Black Americans, for example, have a learned tendency to underreport symptoms,^26^ which can be intensified in the context of an ambiguous question.

Finally, the time frame of reference (i.e., exposure window) can also differentially affect the reporting of SCD across populations. Some questionnaires ask respondents to compare current cognitive functioning to only 1 year ago, while others probe change over much longer time windows, such as going back to high school. Recall of symptoms across time windows can be strongly influenced by cultural factors such as value systems, social norms, and language, which shape whether people focus on the past, present, or future and how they organize their memories and health concerns. Many Western, industrial (monochronic) cultures view time as linear and a valuable resource (“time is money”), prioritizing process events such as schedules and deadlines. Conversely, many Latin American, African, and Middle Eastern (polychronic) cultures view time as flexible and cyclical, prioritizing relationships. In other words, questions that ask about problems with tasks rather than those that interfere with personal relationships may have less resonance in many social groups. Moreover, the salience of linear units of time, such as 3 or 6 months, may be less pronounced in some cultures.

### 2.2 Scientific sampling strategies and measurement approaches are often biased by social practices and political regulations

Recruitment strategies are often grounded in historically discriminatory structural and social factors that have shaped the health and scientific fields, yielding biased samples across socioculturally diverse groups.^27^ Sampling strategies often rely on specific social services and practices to access a given population. The decisions about recruitment strategies directly affect the type of participant targeted for enrollment in the study. The most common consequence of these decisions is the need for more representation of diverse individuals.^28^ Individuals from diverse backgrounds are commonly a small proportion of the samples included in studies. Many studies use convenience samples that do not nearly represent the spectrum of structural characteristics, such as education, setting (e.g., rural, urban), access to services, and socioeconomic status (income), of the larger population. This is true for health outcomes at large, but is especially relevant for the study of SCD, as these factors substantially inform lived experiences and subjective judgments, and are closely related to perceived quality of life.

The selection of individuals for studies can significantly impact the outcomes of interest. Compared to the community, those recruited from clinics may have higher rates of cognitive impairment and may have more robust associations between SCD and future risk of dementia.^29^, ^30^ These effects can be amplified if the recruitment strategy differs across diverse demographic groups. For example, a US study showed that individuals who self‐identified as Black were less likely to be referred by physicians to Alzheimer's Disease Research Centers. Being referred by a physician was associated with increased likelihood of cognitive progression; thus, as the authors concluded, referral sources can bias estimates of cognitive decline across ethnoracial groups.^31^ Similarly, discrepancies between recruitment strategies across ethnic groups have been documented in clinical trials.^28^, ^32^ Future studies should ensure that culturally informed and equitable strategies are implemented, along with procedures for collecting information on who is approached and who agrees to participate in the study.^28^, ^33^ Without adequately addressing these gaps and discrepancies, the literature may present a characterization of SCD that is not representative of populations at large, possibly leading to misinterpretations of the risk that SCD poses for future cognitive decline.

An issue related to, but distinct from, representation is the measurement of sociocultural diversity, particularly sexual and gender diversity, in ADRD studies and, consequently, SCD studies. There is a lack of representation and consistency in measures used to categorize sexual and gender diversity.^34^, ^35^ A study among Alzheimer's Disease Research Centers across the United States found that fewer than 1 in 5 cohorts had documented definitions of “men” and “women,” and that most relied on ambiguous self‐report questions, in which sex and gender are often conflated, to capture these data.^36^ At the same time, studies have shown that there are differences between men and women in terms of how SCD relates to risk of cognitive decline.^37^, ^38^ While the US National Institutes of Health defines sex and gender broadly, most studies rely solely on singular measures of sex/gender identity to independently characterize sex and gender diversity. This typical practice, which offers an incomplete characterization of sex/gender diversity and is combined with normative assumptions about most research cohorts, leads to unmeasured differences across samples. These limitations partially reflect the historical structure of long‐standing cohorts, in which sociocultural variables were not systematically collected. Although more recent studies increasingly attempt to capture gender and sexual diversity, ethical and regulatory frameworks, as well as social acceptance, differ across countries and may limit the collection or accessibility of such sensitive data. As a result, these dimensions remain inconsistently measured and often underdefined in existing datasets.

### 2.3 Limits of construct variables present challenges for validating SCD

To appreciate the clinical value of SCD, many studies have sought to prove that the various instruments designed to capture the intended theoretical concept of SCD are valid (i.e., have good construct validity). A great deal of research also focuses on the criterion, diagnostic, and predictive validity of SCD, showing how it relates to various other constructs, such as cognitive test scores, ADRD biomarker test results, and imaging data that characterize brain degeneration, as well as the likelihood of future cognitive decline. A concern with this approach is that these outcomes share many of the same limitations that impede research on SCD.

First, at present, gold standards for thresholds of clinical impairment and ADRD biomarker positivity have been defined in relatively homogeneous populations. Validation studies have included highly educated White individuals, failing to recognize the importance of diversifying the research population.^39^ This practice has come under increasing scrutiny in ADRD research. For example, the impacts of these practices are causing Black Americans, as one key marginalized social group, to qualify less frequently for clinical trials and as candidates to receive disease‐modifying treatments, compared to their White counterparts.^28^, ^40^ Adding to these concerns is the fact that, in trials of preclinical AD, in which individuals may have biomarkers but not yet show signs of clinical impairment, SCD can be helpful both as a recruitment strategy and as a predictor of clinical progression;^41^ thus, SCD should be tested across diverse social groups to ensure proper validity against criterion variables.

Because reporting SCD in and of itself does not guarantee future cognitive decline, it is important to identify characteristics of SCD that are associated with a heightened risk of progression to dementia. To fully appreciate when SCD might represent the “clinical stage 2” in individuals with positive biomarkers for AD, researchers must consider how biomarkers may vary across disease staging, assessment strategy, patient or participant selection, and sample stratification.^42^ Further, limitations of AD biomarkers can also intersect with sociocultural factors. For example, individuals with chronic kidney disease have higher plasma phosphorylated tau (p‐tau)217, which is becoming the standard blood‐based biomarker for amyloid burden, thereby limiting the utility of plasma p‐tau217 as an AD biomarker in people who have this condition.^43^ Chronic kidney disease disproportionately affects Black individuals compared to White individuals.^44^ The lack of appropriate characterization of the biological AD burden has embedded biases that impact the extent to which SCD is valid in populations other than non‐Hispanic/Latinx White groups. Simultaneously, while current criterion variables may misestimate the prevalence of SCD, clinical criterion variables estimate that the burden of disease is disproportionately higher among socially marginalized groups.^45^, ^46^ Numerous studies have shown that Black and Hispanic/Latinx White older adults are more likely to be diagnosed with MCI or dementia than non‐Hispanic/Latinx White older adults.^47^, ^48^, ^49^, ^50^ The disconnect between the biological burden of AD and the clinical burden of dementia remains an ongoing point of investigation in the AD research community. It raises multiple questions about the appropriateness of AD biological variables as criterion variables for SCD. At the same time, it is imperative to bear in mind that a biomarker for ADRD is not designed to measure SCD or index clinical staging, nor is an SCD instrument designed to detect underlying neuropathology. As these measures cover distinct but, to some degree, related constructs, one must exercise caution when using one as a variable to test the criterion validity of the other.

The differences in rates of MCI and dementia diagnoses have often been attributed to differences in education attainment, school quality,^51^ neighborhood disadvantage,^52^, ^53^ and racial discrimination^54^, ^55^ as determinants that lead to certain groups experiencing disproportionate exposure to risk factors and thus more often meeting clinical criteria.^47^, ^56^ In addition to variable exposure to AD risk factors and systemic disparities in access to services and discrimination in the determination of diagnosis, components of the diagnostic process may also contribute to bias in diagnosis rates. Cognitive testing plays a key role in diagnosis, because without evidence of cognitive impairment on standardized testing, MCI or dementia cannot currently be diagnosed. Cognitive test development suffers from many of the same limitations as AD biomarkers and other diagnostic criterion variables. For example, some tests have been shown to have poor specificity and validity in non‐Hispanic/Latinx White populations without higher education.^57^, ^58^, ^59^, ^60^, ^61^ This could mean that these populations may be classified as having SCD less often when instruments are used that are inadequate to measure their objective cognitive impairment, as they might be considered objectively impaired at lower thresholds than highly educated individuals.

Compounding issues of specificity and validity, the psychometric process of norming, during which test scores are standardized against large samples that are representative of a given population to create interpretable meaning and facilitate comparison, can also bias cognitive data. Ethical and scientific concerns about norming have been raised related to both cognitive and biomarker data.^46^ The implementation of norms and population‐specific cut points also raises challenges for the use of cognitive data. Some studies have shown that including native language^62^ and race or ethnicity^63^ when creating normative data may improve diagnostic accuracy by effectively building for structural influences. However, as recently as the late 2010s, race and ethnicity were collapsed into a single category, which effectively conflates major structural barriers of racialization and language that differently affect social groups. Nonetheless, even this single item of ethnoracial background was reported in only 36% of neuropsychological research studies, and the language of testing was reported in just 20%.^64^ These data are important to report, especially in multilingual contexts in which participants may not be tested in their native or first language, and for the emerging role of language in ADRD.^65^ Furthermore, data from normative studies often do not apply to marginalized individuals.^66^ As a result of these problems with cognitive tests, estimates of SCD prevalence across populations may be inaccurate, either over‐ or underrepresented, depending on the context.

## 3 ASPECTS OF DIVERSITY AND THEIR INFLUENCE ON SCD

Acknowledging the limitations in measuring SCD described above, we now turn to a summary of the literature that investigates explicitly how various aspects of diversity influence the utility of SCD as an early clinical stage of ADRD. In our analysis of the extant literature, we identified themes suggesting that expanding the representation of research participants could address two significant issues. First, a broader representation of social, clinical, and biological diversity improves the generalizability of what is learned about SCD to all people with memory concerns. Second, while the subjective nature of SCD can have idiosyncratic features, it is situated within the structural and social context of groups of people, influencing overall health and cellular function of those groups, and forming the “social exposome,” representing the cumulative external, environmental, and social stressors a person experiences over a lifetime. Understanding the contributors to construct variance can guide the development of a universal, consistent understanding of SCD, helping avoid measuring unrelated constructs and resulting in low measurement precision.

Furthermore, the literature lists differences in the associations between SCD and related outcomes of interest, such as ADRD biomarkers and objective cognitive testing. Some factors modify the overall endorsement or level of SCD as well as its link to cognitive decline and ADRD. For example, confirmation of a complaint by an informant, concerns or worries associated with the complaint, the persistence of a problem over time, the age at which a complaint emerges, and seeking medical help for a given complaint can be related to a higher risk of cognitive decline or diagnostic conversion. Many of these factors have been highlighted as “SCD‐plus” criteria, suggesting an increased likelihood of incipient ADRD.^1^, ^2^, ^67^ Most of these effects have not yet been formally assessed across groups defined by aspects of diversity and vary widely across existing studies. Below, we will describe SCD research focusing on distinct aspects of diversity: race, ethnicity and nationality, sex, gender identity, and sexual orientation. Table 1 summarizes the current literature in these areas.^29^, ^68^, ^69^, ^70^, ^71^, ^72^, ^73^, ^74^, ^75^, ^76^, ^77^, ^78^, ^79^, ^80^, ^81^, ^82^, ^83^, ^84^, ^85^, ^86^, ^87^, ^88^, ^89^, ^90^, ^91^, ^92^, ^93^, ^94^, ^95^, ^96^, ^97^, ^98^, ^99^, ^100^, ^101^, ^102^, ^103^, ^104^, ^105^, ^106^, ^107^, ^108^, ^109^, ^110^, ^111^, ^112^, ^113^, ^114^, ^115^

**TABLE 1:** Overview of prevalences reported across various studies examining aspects of diversity, highlighting the variability in populations, associations with AD outcomes, operationalizations of SCD, and reference frames.

Demographic	Study	SCD prevalence (%)	Association with AD outcomes	Sample was cognitively unimpaired	Single item or scale	Cognitive domains covered	Reference frame	Other dimensions included
**United States**								
**Hispanic/Latinx**	Wooten et al.^68^	11%	—	—	Single item	Memory	Change (1 year)	Medical help‐seeking
	Chapman et al.^69^	—	Yes	Yes	Scale	Memory	Current ability	—
	Corlier et al.^70^	—	Yes	No	Scale	Cognition	Change (10 years)	—
	Rodríguez et al.^71^	—	Yes	Yes	Scale	Cognition	Current ability & change, age‐anchored	Concern
	Ferraro et al.^72^	25%	No	Yes	Single	Memory	Change (2 years)	—
	Eaglesham & Barry^73^	—	Yes	No	Scale	Cognition	Change (2 years)	—
	Zlatar et al.^74^	—	No	No	Scale	Cognition	Current ability	—
	Hall et al.^75^	42%	No	Yes	Single item derived from clinical interview	Cognition	—	Concern
	Tomaszewski Farias et al.^76^	49%	No	Yes	Scale	Cognition	Change (10 years)	—
	Márquez et al.^77^	67%	Yes	No	Scale	Cognition	Change (10 years)	—
**Black/African American**	Wooten et al.^68^	10%	—	—	Single item	Memory	Change (1 year)	Medical help‐seeking
	Abner et al.^78^	21%	Yes	No	Single item	Memory	Change (age‐anchored)	—
	John et al.^79^	24%	Yes	Yes	Single item derived from clinical interview	Memory	Change from previous level	—
	Arvanitakis et al.^80^	32%	Yes	Yes	Two items	Memory	Current ability & change (10 years)	—
	Boggess et al.^81^	25%	Yes	Yes	Single item derived from depression questionnaire and clinical interview	Memory	Current ability	—
	Parisi et al.^82^	—	Yes	Yes	Scale	Memory	Current	—
	Jackson et al.^83^	—	No	Yes	Combined score from multiple scales	Cognition	Current ability & change (10 years)	—
	Sims et al.^84^	—	No	Yes	Scale	Memory	Change (since high school)	—
	Sperling et al.^85^	25%	No	Yes	Scale	Memory	Change (1 year)	—
	Blazer et al.^86^	—	No	—	Single item	Memory	Change	—
	Tomaszewski Farias et al.^76^	35%	No	Yes	Scale	Cognition	Change (10 years)	—
**US Native American**	Wooten et al.^68^	17%	—	—	Single item	Memory	Change (1 year)	Medical help‐seeking
	Luo et al.^87^	13%	—	—	Single item	Memory and confusion	Current ability	—
**Asian American**	Luo et al.^87^	7%	—	—	Single item	Memory and confusion	Current ability	—
	Lee et al.^88^	Korean American: 50% Chinese American: 49% Vietnamese American: 91%	—	—	Scale	Memory	Change (1 month)	—
**Latin America**								
**Brazil**	Borelli et al.^89^	29%	—	Yes	Two items	Memory	Current ability & change (2 years)	—
	César‐Freitas et al.^90^, ^91^	45%	Yes	Yes	Single item	Memory	Change (self‐report), current ability (study partner)	—
	Minett et al.^92^	21%	Yes	No	Two items	Memory	Current ability	Interference with daily functioning
**Chile**	Oyarzún‐González et al.^93^	16%	Yes	No	Single item	Memory	Change	—
	Molina‐Donoso et al.^94^	—	No	Yes	Single item	Memory	Current ability	—
**Mexico**	Miu et al.^95^	53%	Yes	No	Single item	Concentration, Memory	Change (1 month)	—
**Colombia**	Norton et al.^96^	—	Yes	Yes	Scale	Memory	Current ability	—
	Gatchel et al.^97^	—	Yes	Yes	Scale	Memory	Current ability	—
**Sub‐Sahara Africa**	Röhr et al.^98^	51%	—	Yes	Single item	Memory	Current ability	—
**Eastern Asia**								
**China**	Cheng et al.^29^	58%	Yes	—	Scale	Cognition	Change (various time frames)	Worry
	Li et al.^99^	—	Yes	Yes	Single item	Memory	Current ability	Onset, persistence over time
	Chen et al.^100^	—	Yes	Yes	Scale	Memory	Current ability & change (various time frames)	Interference with daily functioning
	Dong et al.^101^	—	Yes	Yes	Scale	Memory	Current ability & change (various time frames)	—
	Wang et al.^102^	—	No	Yes	Scale	Memory	Current ability & change (various time frames)	—
**Japan**	Nemoto et al.^103^	33%	—	Yes	Three items	Cognition	Current ability	Informant
	Goda et al.^104^	2%–27%	Yes	Yes	Three items	Cognition	Current ability	Informant
	Okura et al.^105^	27%	—	—	Three items	Cognition	Current ability	Informant
**South Korea**	Lee et al.^106^	38%	Yes	Yes	Single item	Memory	Change (1 year)	—
**Southeast Asia**								
**Thailand**	Pinyopornpanish et al.^107^	7%	—	Yes	Single item	Memory	Change	—
**Singapore**	Röhr et al.^98^	12%–25%	—	Yes	Single item	Memory	Current ability	—
**Malaysia**	Johar et al.^108^	59%–62%	—	—	Two items	Memory, Concentration	Change (1 month)	—
	Muhamad Nazri et al.^109^	25%	No	No	Single item	Memory	Change	—
	Yap et al.^110^	62%	—	—	Single item	Memory, Concentration	Change (1 month)	—
	Yap et al.^111^	39%	—	—	Single item	Memory, Concentration	Change (1 month)	—
**Indonesia**	Pradono et al.^112^ ^(a)^	14%	—	—	Single item	Memory	Current ability	Informant
**Vietnam**	Meyer et al.^113^	38%–64%	Yes	No	Scale	Memory	Current ability	Worry, interference with daily functioning
**Groups defined by aspects of sexual diversity**								
**Heterosexual, cisgender males**	Flatt et al.^114^	11%	—	—	Single item	Memory and confusion	Change (1 year)	—
**Heterosexual, cisgender females**	Flatt et al.^114^	10%	—	—	Single item	Memory and confusion	Change (1 year)	—
**Gay, cisgender males**	Flatt et al.^114^	11%	—	—	Single item	Memory and confusion	Change (1 year)	—
**Lesbian, cisgender females**	Flatt et al.^114^	17%	—	—	Single item	Memory and confusion	Change (1 year)	—
**Transgender individuals of all sexual orientations**	Flatt et al.^114^	17%	—	—	Single item	Memory and confusion	Change (1 year)	—
**Bisexual, cisgender males and females**	Flatt et al.^114^	18%	—	—	Single item	Memory and confusion	Change (1 year)	—
**LGBT older adults**	Flatt et al.^115^	19%–31%	—	—	Scale	Cognition	Current ability	—

*Note*: “—” denotes that the information was not reported in the study.

Abbreviations: AD, Alzheimer's disease; LGBT, lesbian, gay, bisexual, transgender; SCD, subjective cognitive decline.

^a^ This study only included women.

### 3.1 Race, ethnicity, ancestry, and nationality

Historical structures of subjugation and bias have had different targets (e.g., race, ethnicity, nationality, immigration status, language) across the world. In the United States, the history of slavery and subsequent segregation has caused substantial lingering biases based on race. Historical systems of social stratification and discrimination vary across regions but can shape health outcomes through structural and interpersonal biases. These dynamics influence access to health care, the interpretation of symptoms, and the reporting of cognitive concerns. As a result, sociocultural characteristics such as race, ethnicity, nationality, and migration history may influence both the prevalence of SCD and its associations with dementia risk and biomarkers.

Cultural, social, and environmental dynamics are modifiers of self and informant reports,^115^ which remains a key gap in the literature. For example, a person's comfort with reporting and expressing their own difficulties, or those of a loved one, can vary depending on cultural norms. In the context of SCD, these dynamics may lead not only to people underreporting but also to differences in how cognitive concerns are interpreted, valued, or acted upon in clinical settings. Still, relatively few studies even report the participant characteristics needed to understand the contexts in which the study was conducted, and in which an individual participated.

Ten years ago, only 5 of 53 studies on SCD assessed in a meta‐analysis reported the race/ethnicity of study participants,^116^ making it impossible at that time to assess ethnoracial influences on the relationship between SCD and cognitive outcomes. Today, many studies, particularly those from the United States, report information on race and ethnicity, although many are still composed mainly of White, non‐Hispanic participants. There is some evidence that SCD may be more common among Asian and Black/African Americans,^97^ and Hispanic/Latinx and Native Americans,^86^ compared to their non‐Hispanic White counterparts. Furthermore, in the United States, studies have shown that SCD is differentially associated with AD biomarkers and future cognitive impairment in different ethnoracial groups.^68^, ^117^

While the interrogation of the influences of systemic biases (e.g., linguistic, cultural, and ancestral lineage) on SCD remains largely unexamined, promising national data are emerging worldwide. In Latin America, efforts to characterize SCD have recently emerged, and nationally representative cohorts have been established in Peru, Chile, and Ecuador to document the natural history of SCD in these populations.^118^, ^119^, ^120^ In convenience samples in Colombia, investigations have been initiated to understand the associations between SCD and AD biomarkers.^95^, ^96^ In Brazil, SCD is robustly associated with self‐identified race, where Brown/Pardo and Black individuals have an increased risk of SCD compared to White individuals.^88^ This has been attributed to disparities based on social background and unequal access to health care.

In Eastern and Southern Asia, one of the most diverse regions on Earth, home to thousands of distinct cultural identities, most studies are emerging from China,^29^, ^98^, ^99^, ^121^ which contains 56 ethnic groups and >300 living languages. There remains little work on the associations between SCD and ADRD clinical characteristics and biomarkers across these regions. In India, research on SCD and these characteristics also remains limited.^122^, ^123^, ^124^ The research to date on SCD has focused on distinct clinical groups, with nearly half of older adults with typical objective test scores in urban South India reporting cognitive deficits.^124^ Importantly, South India has the country's most advanced medical infrastructure and some of the best health outcomes in South Asia. Only more recently have North, Central, and Northeast India begun to experience public and private investment in healthcare infrastructure, underscoring the bias in research toward groups benefiting from dominant social structures and the influence of “universal” standards or exposures that are actually specific to privileged demographics.

Relatively little work on SCD has emerged from the African continent due to limited resources allocated to cognitive research, though this is slowly increasing. One study from Malawi found that women had lower cognitive performance than men, contrary to what is often reported in studies from high‐income countries.^125^ Another study using data from Ghana and South Africa, as well as from low‐ and middle‐income countries elsewhere, showed that SCD was higher among people with lower food security.^126^ A global survey study that included several countries in Africa, including Burkina Faso, Ethiopia, Ghana, Ivory Coast, Kenya, Malawi, Mali, Mauritania, Namibia, Republic of Congo, Senegal, South Africa, Swaziland, Tunisia, Zambia, and Zimbabwe, found that depressive symptoms relate to SCD,^127^ although the risk of future dementia in this context is yet to be investigated. At the same time, it is essential to consider the broader context in which individuals are aging. For example, data from sub‐Saharan Africa indicate that life expectancy is lower than in most low‐ and middle‐income countries elsewhere,^128^, ^129^ and that the adverse effects of aging occur earlier in the life course.^130^, ^131^

As the public's knowledge about dementia and dementia risk is growing, the emergence of national dementia or AD plans is a promising development that could help improve the identification of SCD and its causes in the context of ADRD. These plans help support efforts to promote early diagnosis and implement caregiver supports, increase resource allocation, and reduce the stigma surrounding dementia and specific subtypes of ADRD. As of 2025, >50 countries have such plans.^132^

Factors that may drive or increase risk of SCD differ across global and local regions. In Brazil, a study found that hearing loss is the most robust predictor of SCD, followed by low education and psychological distress. In China, clinical significance is often tied to social network factors, such as living alone or a lack of close friends, as well as to vascular health.^29^ In Malaysia, one study found that social capital and chronic conditions, such as arthritis, significantly influence the impact of SCD on an individual's self‐rated health.^107^ In low‐ and middle‐income countries in general, food insecurity has been found to be significantly associated with higher subjective cognitive concern rates, partially mediated by depression and sleep disturbances.

### 3.2 Socioeconomic status

The socioeconomic drivers of SCD differ globally based on countries’ income levels, education infrastructure, population demographics, and regional stressors. The impact of these factors varies substantially between high‐income and low‐ and middle‐income countries. In low‐ and middle‐income countries, drivers of SCD are often related to extreme poverty and lack of necessities. For example, in Vietnam, material hardship, defined as a lack of access to resources like clean water, food, and housing, was a stronger correlate of memory concerns than in Western samples.^112^ A study of older adults in six low‐ and middle‐income countries found that severe food insecurity is associated with significantly worse cognitive concerns, a relationship that is partially mediated by the psychological stress and depression caused by the inability to access adequate food.^126^ In Malawi, specific wealth indicators such as having a metal roof predict higher baseline cognitive levels.^126^ Similarly, in Mexico, individuals in the lowest income quintile show significantly poorer cognitive function than those in higher income brackets.^94^

### 3.3 Education and literacy

Schooling and education are major social structures and can reflect a lifelong process of acquiring knowledge, skills, and habits through learning, which is hypothesized to build cognitive reserve that protects the brain. Consequently, education attainment is one of the most significant global drivers of SCD. Global studies of SCD demonstrate that its influence is nuanced. In many low‐ and middle‐income countries and emerging economies, such as Brazil and China, improved access to schooling may reflect stronger social infrastructure that helps protect cognition.^133^ Low education attainment is also a robust predictor of SCD.^88^, ^97^ Epidemiological data from Brazil indicate that individuals with SCD have significantly lower mean years of education compared to those who are cognitively unimpaired.^88^ This regional trend is mirrored in China, where low education attainment has been associated with increased memory‐related concerns.^29^, ^134^ In India, higher education attainment is significantly associated with fewer memory concerns and better objective cognitive performance.^124^ Specifically, research in South India found that individuals who completed higher secondary school were more likely to be cognitively normal, whereas those who did not complete their education were more likely to be in the cognitive impairment group.^124^

Paradoxically, in some contexts, the role of SCD in predicting future cognitive decline is stronger in highly educated groups.^29^, ^135^ A possible explanation is that individuals with more schooling have greater cognitive reserve, allowing them to tolerate greater brain pathology before showing deficits on objective tests.^29^, ^107^, ^135^ Consequently, in these populations, subjective concerns may reflect subtle, recent changes due to underlying neurodegeneration that standardized screening tools are not yet sensitive enough to detect.^29^, ^135^

In low‐literacy contexts such as rural Malawi, schooling increases the probability of consistently high performance on cognitive assessments, yet cognitive decline often begins at younger mid‐adult ages (45–55 years) than in high‐income countries.^125^ Studying SCD, objective cognitive performance, and schooling in low‐ and middle‐income countries may offer major advances to the scientific understanding surrounding the influence of social infrastructure, environmental exposures, and neuroprotective activities on SCD, with the potential to offer novel insights into the distinct effects of schooling and education and their correlates that can reduce known dementia risk factors.

### 3.4 Rurality and urbanity, and social context

Higher SCD prevalence has been reported in rural populations compared to urban ones in China and Mexico.^29^, ^94^ These associations are likely attributable to lower levels of schooling, lower socioeconomic status, higher rates of unhealthy lifestyles, and delays in health‐care delivery due to limited access. Conversely, a study in India describes how the shift from joint family systems to nuclear families and the migration of children overseas have left an increasing number of older individuals at risk of social isolation, which has downstream effects on the experience and reporting of SCD.^124^ More research is needed to fully understand the effects of isolation and loneliness on SCD.

### 3.5 Sex, gender identity, and sexual orientation

Considering sex and gender identity, which refers to the mixed influence of social and biological factors on identity,^136^ studies to date have focused almost exclusively on the binary identities of male and female.^137^ There is little available data on SCD among individuals who identify along the lesbian, gay, bisexual, transgender, intersex, and non‐binary continuum. Developing knowledge in this area will help clinicians better address cognitive concerns among individuals in these communities, as well as understand how sex, gender, and sexual orientation influence the relationship between cognitive concerns and underlying AD pathology and/or risk of future cognitive impairment. In doing so, it will be essential to address how access to health care, interpersonal interactions, and social stressors may influence the conduct of SCD research with these groups, participation in this research, and the knowledge that emerges from such studies, given that social acceptance and treatment of groups defined by sex and gender identity and sexual orientation can differ based on social norms.

Moreover, epidemiologic studies on the differences between men and women in SCD prevalence rates and risk estimates are inconsistent.^36^, ^134^, ^138^ Some studies have shown that women with SCD decline faster than men^37^ and have a greater amyloid beta deposition.^139^ Some evidence also suggests that women have better memory awareness, defined as the discrepancy between subjective memory complaints and objective memory performance, and thus, their appraisals of SCD might be more accurate.^140^ Studies investigating the risk that SCD poses for future decline should thus incorporate factors such as gender identity, socioeconomic status, and cultural background, which can significantly influence both individual‐ and group‐level perceptions of cognitive decline and their relation to cognitive progression.^141^ Moreover, including measures of gender‐stereotyped behavior and gender role congruence may offer new data on how differences in appraisal may correlate with cultural reporting styles of SCD.^142^

Finally, clinicians can exhibit unconscious sex or gender biases toward patients or caregivers, who are primarily women, and might be heightened at certain sociodemographic intersections, such as those of race and ethnicity.^143^, ^144^ Particularly given the subjective component of SCD, it is crucial to conduct studies that can help account for the effects of observer bias and interpersonal interactions, including those related to gender and other social characteristics, on the SCD construct. Doing so will improve our understanding of the extent to which observed differences in SCD reflect underlying cognitive phenomena versus social and interpersonal factors that shape its assessment.

## 4 MULTIFACTORIAL PRESENTATIONS OF THE EXPRESSION OF SCD: INFLUENCE OF SOCIAL, STRUCTURAL, PSYCHOLOGICAL, AND CULTURAL FACTORS

Prevalence estimates for SCD vary widely, ranging from 5% to >80%. Globally, SCD prevalence is generally higher in older age groups, though studies in Chile and Thailand have documented significant prevalence in younger adults (ages 30–65).^92^, ^106^ Large‐scale studies also indicate that SCD is more prevalent in low‐ and middle‐income countries than in high‐income countries. For instance, prevalence in Brazil has been estimated at ≈30%,^88^ in China at almost 60%,^29^, ^145^ and in the United States at only 11%.^146^

There are many interrelated reasons why prevalence estimates differ so much. Factors such as geographic region, age group, context, criteria used to define SCD, and intercultural differences in reporting styles all contribute to differing estimates. Further, a major source of differences in prevalence is the setting in which SCD is assessed: In population‐based studies, prevalence is much lower than in memory‐clinic patients.^30^ Below, we examine how various social, structural, and cultural factors influence the expression of SCD and, by extension, its prevalence estimates and clinical implications.

### 4.1 Structural and social determinants of health

Structural and social determinants of health are broadly defined as the environments and conditions in which individuals live, work, and age. Core determinants, including economic stability, access to health care, schooling, and neighborhood and community‐built environments, are known to influence cognitive aging and ADRD outcomes.^19^, ^147^, ^148^ Findings from the limited number of studies that have been conducted in this area support the idea that individuals who experience more structural and social barriers also have higher rates of SCD, consistent with previous literature on the negative impact of structural and social determinants of health on risks of cognitive impairment and AD dementia.^19^ Still, associations between structural and social factors and SCD can be complex and multifaceted. For instance, some studies have highlighted that the prevalence of SCD is higher in those with lower levels of formal education.^149^, ^150^ Still, others have shown that SCD in individuals with higher education attainment is a better predictor of AD dementia risk and pathological burden.^135^ Emerging research on systemic oppression shows that racialized experiences, such as access to lower quality schools, can alter aging outcomes, further potentially confounding associations between SCD and ADRD outcomes.^36^

Conceptual work is needed to guide the research and synthesize the information garnered in these studies. For example, as Stites and Velocci^137^ describe, while many US studies have examined sex and gender differences in SCD, they have not attempted to model the specific contributions of social, structural, or biological factors to the observed differences. A unified approach is needed to support the longer term goal of population‐level intervention to reduce SCD and its consequences, which requires understanding its specific typologies, mechanisms, and pathways.

Globally, studies are also lacking at a critical intersection between race on the one hand, and sex and gender on the other. One review that included mostly US‐based samples estimated that ≈ 20% of sex‐ or gender‐minoritized groups are racially minoritized.^151^ This may exacerbate known disparities in ADRD related to both race and ethnicity^47^ and sex and gender,^152^ placing specific subgroups at greater risk. In fact, evidence suggests that sex‐ and gender‐minoritized individuals who are from racially or ethnically minoritized groups report higher rates of SCD.^113^, ^114^, ^153^

Intersectional studies may offer the opportunity to consider how racialized and gendered experiences, including experiences of identity‐based oppression, may compound and jointly influence SCD. For instance, experiences of gendered racism across the lifetime were associated with having more cognitive concerns among a sample of older Black women, and symptoms of depression and coping mediated this relationship.^154^ In addition, Black sexual minority women^155^, ^156^ and Black women with disabilities^157^ experience intersectional discrimination that is associated with an elevated risk of cognitive concerns.

### 4.2 Sociocultural and psychological factors

Closely related to, but distinct from, structural and social determinants of health are sociocultural and socio‐psychosocial factors, which are key determinants of health arising from how social groups and individuals within those groups experience health care and health conditions, including SCD. They pertain to how people think about and use their cognition, and to the social norms that guide how individuals interpret their own cognition and health experiences, such as whether they admit to having difficulties and seek health care, and the type of help they might seek. While structural and social determinants of health focus on systemic factors (i.e., barriers to health care arising from problems within the system), sociocultural and psychological (e.g., psychosocial, psychocultural, and socio‐psychological) factors emphasize how cultural beliefs, social conventions, and social processes impact certain groups (i.e., how groups act and react when excluded or included, and dismissed or valued by health care) and members of certain groups. In this section, we briefly introduce sociocultural factors and psychological processes related to perceptions of aging, ADRD stigma, and cognitive biases, as they are relevant to SCD.

There has been limited research exploring potential differences in aging self‐perceptions and reporting of SCD across racial and ethnic groups. However, studies have consistently found that perceptions of aging are linked to the overall health and well‐being of older individuals.^158^ Because SCD is commonly probed through study partner–completed questionnaires, the relationship between the participant and their study partner may influence the determination of SCD. Indeed, dyadic differences (i.e., differences between the participant and study partner) in the reporting of SCD have been described.^159^ More research is needed to understand how variation in relationships and relationship dynamics, which both can vary across cultures and social contexts, may impact determinations of SCD and the well‐being of persons with SCD. Social position, cultural context, and other characteristics can impact SCD research and clinical outcomes by influencing who serves as a participant's study partner.^115^, ^160^ As such, global variation in family structure stemming from premature mortality, policies that dictate state recognition of relationships, and differences in social expectations of gendered power structures may all pose challenges to SCD research and the clinical translation of SCD‐related findings.

Early work on perceptions of aging has indicated that White individuals often maintain positive self‐perceptions of aging,^161^ a trend that has continued into the present day.^162^ However, those who tend to hold negative attitudes and beliefs about aging have worse health outcomes, including SCD.^163^, ^164^ Across various cultures, the decline in cognitive abilities, particularly memory, is a relatively common and expected part of the aging process.^165^ The extent or threshold at which cognitive difficulties and eventual impairments are considered, along a continuum from a normal part of healthy aging to a sign of an underlying disease, may vary across cultures. A US study found that approximately half of the adult public believed that memory problems are a typical part of normal aging, and those individuals were more likely to dismiss SCD.^166^

The extent to which disease, disability, and ADRD are stigmatized can impact patients’ disclosure of SCD to clinicians and participation in SCD research. In some African and Hispanic cultures, ADRD can be viewed as a curse or punishment, witchcraft, or a sign of mental illness. Additionally, stereotype threat, which refers to a person's worry about conforming to a negative stereotype, has been shown to affect marginalized social groups differentially. Black Americans have been shown to fare worse on cognitive testing due to stereotype threat.^167^ To date, it is not well understood how stigma and stereotype threat may shape a person's reporting of their memory, and if they can modify the associations between SCD and ADRD criterion variables.

Research on health disparities shows that cognitive biases are correlated with individuals’ symptom reporting.^81^, ^82^ Black Americans as a group are less likely than their White counterparts to report symptoms to health‐care providers,^26^ and their symptoms are more likely to be dismissed.^168^ The learned behaviors of minoritized groups can reflect responses to cognitive biases among health‐care providers, which can operate alongside social and structural determinants of health. Women also face biases in health care, where providers more often dismiss their symptoms than those of their male counterparts. At the same time, sex‐ and gender‐minoritized groups are less likely to access care,^169^ even in the absence of social and structural barriers, due to factors including high rates of internalized stigma, fear of discrimination, and previous negative experiences with providers. Thus, variance in SCD may correlate with disclosure or normative assumptions about social identities, as well as with help‐seeking behaviors,^170^, ^171^ and may be influenced by access and prior experience rather than solely by disease pathology. The divergence between SCD in clinical and community settings may, in part, be explained by psychological factors as well as structural and social inequities.

## 5 OVERCOMING THE BARRIERS TO ESTABLISH THE WORLDWIDE CLINICAL UTILITY OF SCD

Based on the literature summarized above, we offer several recommendations to help the SCD field overcome barriers that hinder the establishment of the clinical utility of SCD across the globe. Table 2 lists several barriers and their associated suggested action steps to overcome them. In the text below, we discuss a selection of these action steps in more detail. Most of the recommendations below are ready to be implemented into research and clinical practice at present, particularly if researchers and clinicians prioritize addressing aspects of diversity. However, we also acknowledge that further developments in our understanding of SCD may be a prerequisite for realizing downstream methodological changes.

**TABLE 2:** Identified barriers and recommendations for the advancement of research on SCD.

Barrier	Action steps
Lack of uniformity in SCD definitions and measurement	− Consistently distinguish between SCD as stage 2 of the ADRD clinical continuum (“SCD‐plus”) and subjective cognitive changes that may occur in other contexts, including later disease stages, other diseases, and healthy aging. − Ensure that well‐validated SCD measures are used that are appropriate for the research question and population, paying attention to (1) coverage of cognitive domain, (2) inclusion of relevant time references, and (3) proof of measurement invariance across relevant groups − Acknowledge the limitations of standards used to investigate the relationships between ADRD markers and SCD. − Support rigor and reproducibility in SCD investigations by documenting social, structural, and psychological determinants of study outcomes.
Limits to criterion	− Determine criterion variables that are invariant to factors outside of SCD and its diagnosis. − Continue work on developing culturally diverse norms of cognitive tests and use caution in deploying current norms and cut points. − Use tests that have shown measurement invariance across relevant groups. − Scrutinize criterion variables: Clinical and pathological disease staging reflects the commonly used gold standard against which SCD is compared to assess its validity as an early clinical indicator of ADRD. These outcomes do not measure the exact same thing and thus affect the understanding of SCD if used inappropriately.
Measurement	− Deploy similar methods in socioculturally and socioeconomically varied samples. − Interrogate population‐level variation in SCD to identify components in the multifactorial pathway of SCD.
Lack of adequate representation and sampling strategies	− Use community‐based recruitment strategies to help overcome health and health‐care disparities. − Conduct rigorous studies in groups with intersectional identities and experiences. − Conduct and highlight studies from outside the United States and Europe. − Increase representation of people who identify along the lesbian, gay, bisexual, transgender, intersex, and non‐binary continuum, as well as in women and men from varied socioeconomic backgrounds.
Lack of social and cultural validation and considerations	− Conduct qualitative research studies to qualify the experience of SCD across differing sociocultural characteristics. − Include persons with lived experiences in the conduct of research on SCD. − Undo historical wrongs and marginalization by addressing sample biases. − Investigate cultural, social, and environmental dynamics as modifiers of self‐ and study partner reports.
Achieving goals of improving well‐being of adults with cognitive concerns and advancing methods for early diagnosis and treatment of ADRD	− Investigate how the experience of cognitive decline and reporting of cognitive concerns are affected by aspects of diversity. − Identify factors that contribute to SCD and to variation in the reporting of cognitive concerns. − Investigations of SCD in the context of dementia and comorbid conditions to understand the shared underlying biology implicate operation of a network rather than any specific pathway in the causal mechanism. − Categorize factors that modify the overall endorsement and level of SCD as well as its link to cognitive decline and ADRD. − Interrogate sociocultural, socioeconomic, and sociopsychological factors that can affect how social groups experience SCD. These include differences in aging self‐perceptions (e.g., notions of what a “normal” aging process looks like), attitudes, stigma and stereotype threat, and reporting SCD across different racial and ethnic groups.

Abbreviations: AD, Alzheimer's disease; SCD, subjective cognitive decline.

### 5.1 Explicitly and precisely delineate SCD and its measurement

Important work has been done to identify measurement diversity,^22^ harmonize SCD questionnaires,^20^ and identify professionals’ perspectives on SCD measurement,^172^ yet clinical practice trails behind. While one size will most likely not fit all for SCD measurement, a better global alignment in how SCD is defined, assessed, and contextualized will improve the generalizability of research findings to broader populations and strengthen the construct's rigor.

First, we recommend that a distinction be made between SCD as the second (early) clinical stage in people who are on the AD continuum, according to the NIA‐AA biological framework, and SCD as self‐perceived cognitive changes that may occur in other contexts, including later stages of ADRD, other (non‐ADRD) diseases, environmental stressors, and healthy aging. The former construct is captured by what is referred to as “SCD‐plus,” a designation for individuals experiencing subjective cognitive concerns who meet several specific criteria, including age > 60, recent onset of SCD, worry, and medical help‐seeking.^1^ It has been shown that individuals with SCD‐plus are more likely to show subsequent objective cognitive decline and are more likely to show amyloid pathology.^173^ Conflicting findings imply that more research is needed to fully appreciate how SCD (in the general sense of experiencing cognitive concerns) is specifically linked to ADRD.

Next, it is important that researchers use well‐validated SCD measures. One single instrument that serves all purposes of SCD‐related research is not currently available, and may not even be desirable. When selecting instruments, it is crucial to consider the goal, which may include understanding impacts on quality of life, predicting cognitive impairment, and identifying causes of (self‐perceived) cognitive decline. Differences in (1) the coverage of multiple cognitive domains, (2) the use of relevant time references (e.g., comparing current self‐perceived cognitive functioning to 1 year ago, 5 years ago, or to teenage years), and (3) proven measurement invariance across relevant groups (e.g., defined by sex or gender identity, nationality, race, ethnicity, etc.), impact the measure's sensitivity, reliability, and construct validity.

Third, limitations in the standards used to distinguish SCD as an early marker of ADRD from SCD as a cognitive concern that emerges with aging in persons without known illnesses need to be acknowledged and addressed in the scientific discussion. In the absence of these explicit discussions, the current approach introduces biases into research and exacerbates downstream effects on clinical practice. One way to make this distinction more explicit is to use the term “SCD‐plus” to refer to SCD in the context of preclinical ADRD.

Last, it will ultimately be important to determine criterion variables that are invariant to factors not germane to the condition of SCD, its diagnosis, and its treatment. However, at this point, such variables may not be readily available as more work is needed to understand SCD's position in the ADRD and aging paradigms.

### 5.2 Increase efforts to study aspects of diversity in relation to SCD

We recommend that more attention be given to understanding how experiences of cognitive decline and the reporting of cognitive concerns are affected by aspects of diversity. Further research is needed to understand the multiple factors (e.g., biological, social, psychological, and structural) that can influence the experience and expression of SCD, as well as its association with ADRD outcomes. These factors include differences in aging self‐perceptions (e.g., notions of what a “normal” aging process looks like), attitudes, and stigma. To advance research in this realm, it will be crucial to characterize samples by the factors that modify the overall endorsement and level of SCD, as well as its link to cognitive decline and ADRD, such as cognitive status (e.g., distinguishing between those who are objectively impaired and those who are unimpaired), diagnosis (including etiological or syndromic diagnoses), and the relevant disease biomarker levels (when assessed). When researchers document and report the social, structural, and psychological determinants of study outcomes, this increases the rigor and reproducibility of the investigations.

In pursuing this line of study, it is essential to broaden the sociocultural composition of research samples. When recruiting participants, researchers should be cognizant of the sociocultural composition of the population in the area where the research is conducted and strive to align the research sample with the local population. Concurrently, because subjective experiences may be shaped by sociocultural and individual factors, approaches that allow for contextualized or individualized interpretation are also worth considering. Qualitative research that more thoroughly probes people's experiences may help clarify the experience of SCD across differing sociocultural contexts.

The field needs to continue developing culturally diverse norms for cognitive tests and use caution when deploying current norms and cut points. Furthermore, the criterion variables that are most often used to validate SCD require further scrutiny in the context of diversity. When construct and criterion validity of SCD across measures is examined more carefully in diverse sociocultural cohorts, it will become clear whether SCD measures tap into the same construct across different groups. As an extension, further research is needed to develop culturally appropriate measures that achieve construct invariance across groups.

Finally, it is recommended that the influence of relevant aspects of diversity be investigated in statistical analyses, for example, by including them as covariates and using data‐reduction techniques to identify primary drivers of effects. Provided that a study has sufficient range in a given aspect of diversity, it may also be important to use multilevel models, interaction terms, or stratified analyses. Multilevel models can adjust for hierarchical nesting of data, for example, within sex or gender identities, nationalities, or race or ethnicities. Interaction terms can reveal potential modifying effects, and stratification can be used to investigate associations in separate sub‐groups. However, for reliable estimates, it is important that the distribution of participants is not severely skewed toward one group.

### 5.3 Improve representation and sampling strategies

The field at large has the responsibility to undo historical wrongs and marginalization by addressing sample biases. To help overcome health and health‐care disparities, we recommend that researchers use community‐based recruitment strategies; conduct rigorous studies in groups with intersectional identities and experiences; and increase representation of people who identify along the lesbian, gay, bisexual, transgender, intersex, and non‐binary continuum, as well as in women and men from varied socioeconomic backgrounds. Inclusion of sex‐specific biological markers, while not specific to SCD, may nonetheless help support understanding of how SCD relates to other sex‐specific experiences. Including persons with lived experience in research on SCD may help advance understanding of how SCD is affected by the progression of ADRD and the daily impact of living with SCD, which is essential to ensuring a patient‐centered approach to caring for persons with SCD. Perspectives from diverse individuals living with SCD are needed to better classify its different types, identify factors that modify it, and understand the core ways in which SCD impairs well‐being and quality of life.

As a global field, we need to continue expanding the conduct of studies outside the United States, Europe, and other high‐income countries. As resources allotted to ADRD research increase in low‐ and middle‐income countries, we suggest that evidence‐based research methods be deployed across socioculturally and socioeconomically varied samples. In addition to reporting on well‐characterized samples, it is essential to undertake analyses that aid in identifying the components and timing of SCD's multifactorial pathway.

## 6 CONCLUSION

In conclusion, the historical patterns of dismissing or misinterpreting cognitive reports from women; individuals who have non‐normative gender identities and sexual orientations; and members of racially, ethnically, or ancestrally minoritized communities have had lasting consequences for health outcomes.^26^, ^174^, ^175^ This problematic history is compounded in the field of ADRD, as the stigma of dementia can interact with other social biases.^176^ ADRD also has a high social burden that can exacerbate systematic exclusion and other consequences of low socioeconomic resource settings, which members of minoritized communities disproportionately face. Combined with the ambiguous, non‐specific nature of cognitive concerns and common lack of clear definition of SCD, these factors complicate the measurement of SCD and the study of its place as a preclinical marker of ADRD. Due to a combination of factors, the biomedical research samples used to study SCD in ADRD are predominantly composed of individuals from a small, privileged group that does not adequately reflect the global aging population. Acknowledging this fundamental issue is the first step toward improving SCD research and, eventually, clinical outcomes for everyone at risk of cognitive impairment.

This paper offers a critical analysis of social and cultural diversity in research on SCD. An examination of the current landscape of SCD research conducted across diverse sociocultural samples of older adults offers a lens for identifying gaps and providing recommendations for future investigation. The inclusion of a broad range of sociocultural groups is essential to adequately understand SCD and its clinical implications, which may include an increased risk for dementia and reduced quality of life. Vital work in this field is already under way, but even more resources need to be dedicated to the study of SCD, particularly in middle‐ and low‐income countries, to fully understand SCD and its place within the clinical continuum of ADRD. As much remains unknown, addressing questions about how aspects of diversity influence the experience and reporting of SCD offers a useful roadmap for dismantling health disparities and for discovering pathways of SCD to understand how it operates within the paradigms of ADRD diagnosis, treatment, and, ultimately, prevention.

## CONFLICT OF INTEREST STATEMENT

Mark A. Dubbelman, Tanisha G. Hill‐Jarrett, Silvia Chapman, Martina Azar, Tyler R. Bell, Mark A. Bernard, Carolina Boza‐Calvo, Anthony Q. Briggs, Heather Cuevas, N. Maritza Dowling, Bernadette A. Fausto, Carol E. Franz, Joyla A. Furlano, Christopher Gonzalez‐Corona, M. Aaron Guest, Veer Bala Gupta, Vivek K. Gupta, Daija Jackson, Jillian L. Joyce, Michael R. Kann, William S. Kremen, Athene Lee, Jairo E. Martinez, Arjun V. Masurkar, Maia A. McLin, Elisa de Paula França Resende, Zahra Rahemi, Brianca C. Renfro, Márlon Juliano Romero Aliberti, Shaina Shagalow, Juliana N. De Souza Talarico, Jean François Trani, Carol Van Hulle, Leah Waltrip, Stephen Ojiambo Wandera, Mônica Sanches Yassuda, Davide V. Moretti, Elke Butterbrod, Katherine A. Gifford, Sietske A.M. Sikkes, and Shana D. Stites have no relevant disclosures to report. Ganesh M. Babulal is funded by the National Institutes of Health (NIH)/National Institute on Aging (NIH/NIA) grants as PI (R01AG089700, R01AG080469, R01AG067428, R01AG068183, R01AG067428, R13AG096982). Omonigho M. Bubu is supported by the NIH through the following grants: K23AG068534, R01AG082278, RF1AG083975; Alzheimer's Association grant AARG‐21‐848397, Alzheimer's Association/Michael J. Fox Foundation/CurePSP SCN‐25‐1474727 and BrightFocus Foundation A2022033S and is a member of the ResMed Sleep Science Advisory Group, the Alzheimer's Association International Research Grant Program Council, the American Thoracic Society Conference Program Planning Committee, the American Academy of Sleep Medicine Scientific Review Committee, SLEEP conference Program Planning Committee, Sleep Research Society Diversity, Equity, Inclusion, and Accessibility Committee, and Chair of the Low and Middle Income Countries Work Group in the Diversity and Disparities PIA of ISTAART. He also serves in the editorial board of the *Alzheimer's & Dementia* journal, *Current Sleep Medicine Reports*, and the *Journal of Alzheimer's Disease*. Darlingtina K. Esiaka has received grants from the Alzheimer's Association. Fabricio Ferreira de Oliveira is supported by FAPESP—The State of São Paulo Research Foundation, grant #2015/10109‐5. Andrea Slachevsky is partially supported by ANID/Fondecyt/1231839, Alzheimer Association (ALZ‐RWD‐26‐1466627), and NIH‐NIA (R01AG075775, R01AG083799, 2P01AG019724); and the Multi‐partner Consortium to Expand Dementia Research in Latin America (ReDLat), which is supported by the Fogarty International Center and the National Institutes of Health, the National Institute on Aging (R01AG057234, R01AG075775, R01AG21051, and CARDS − NIH), Alzheimer's Association (SG‐20‐725707), Rainwater Charitable Foundation's Tau Consortium, the Bluefield Project to Cure Frontotemporal Dementia, and the Global Brain Health Institute. Charles C. Windon has received grants from the NIH‐NIA (K23AG093166) and Alzheimer's Association. Elizabeth Kuhn has received postdoctoral grants from Fondation Alzheimer (France, 2025‐28); Helmholtz AI Corporation Unit (Germany, ZT‐I‐PF‐5‐163; 2023‐25), and a prize from Fondation Planiol & Inner Wheel (France, Young career researcher for a publication, 2026). Raphael M. Castilhos has received grants from the Alzheimer's Association. Rachel L. Nosheny has received grants paid to her to institution from NIH, Alzheimer's Association, California Department of Public Health, Genentech, and Lilly. None of the funders or other affiliations of any of the authors had any role in the conception or preparation of this manuscript. Author disclosures are available in the Supporting Information.

## Supporting information




**Supporting Information**: alz71779‐sup‐0001‐SuppMat.pdf
